# Influence of PB2 host-range determinants on the intranuclear mobility of the influenza A virus polymerase

**DOI:** 10.1099/vir.0.031492-0

**Published:** 2011-07

**Authors:** Ágnes Foeglein, Eva M. Loucaides, Manuela Mura, Helen M. Wise, Wendy S. Barclay, Paul Digard

**Affiliations:** 1 Division of Virology, Department of Pathology, University of Cambridge, Tennis Court Road, Cambridge CB2 1QP, UK; 2 Imperial College, London, UK

## Abstract

Avian influenza A viruses often do not propagate efficiently in mammalian cells. The viral polymerase protein PB2 is important for this host restriction, with amino-acid polymorphisms at residue 627 and other positions acting as ‘signatures’ of avian- or human-adapted viruses. Restriction is hypothesized to result from differential interactions (either positive or inhibitory) with unidentified cellular factors. We applied fluorescence recovery after photobleaching (FRAP) to investigate the mobility of the viral polymerase in the cell nucleus using A/PR/8/34 and A/Turkey/England/50-92/91 as model strains. As expected, transcriptional activity of a polymerase with the avian PB2 protein was strongly dependent on the identity of residue 627 in human but not avian cells, and this correlated with significantly slower diffusion of the inactive polymerase in human but not avian nuclei. In contrast, the activity and mobility of the PR8 polymerase was affected much less by residue 627. Sequence comparison followed by mutagenic analyses identified residues at known host-range-specific positions 271, 588 and 701 as well as a novel determinant at position 636 as contributors to host-specific activity of both PR8 and Turkey PB2 proteins. Furthermore, the correlation between poor transcriptional activity and slow diffusional mobility was maintained. However, activity did not obligatorily correlate with predicted surface charge of the 627 domain. Overall, our data support the hypothesis of a host nuclear factor that interacts with the viral polymerase and modulates its activity. While we cannot distinguish between positive and inhibitory effects, the data have implications for how such factors might operate.

## Introduction

The natural reservoir of influenza A viruses is waterfowl, but a wide range of avian and mammalian hosts can be infected (Webster *et al.*, 1992). Influenza strains adapt to a specific host, and only rarely does a virus infect and transmit efficiently in a new species. For humans, this last happened in 2009, with a swine-origin virus that initiated the first pandemic of the 21st century (Taubenberger & Kash, 2010). Since 1997, H5N1 avian influenza viruses have caused sporadic, severe infections in man. However, sustained human transmission resulting in a ‘bird-flu’ pandemic has not yet occurred, for reasons that are unclear but presumably reflect the difficulty of successfully crossing the species barrier.

Influenza A virus has a negative-stranded RNA genome whose eight vRNA segments encode up to 12 proteins (Wise *et al.*, 2009). The virus encodes its own RNA-dependent RNA polymerase, which transcribes and replicates the virus genome (Neumann *et al.*, 2004). The polymerase complex (3P) is a heterotrimer of PA, PB1 and PB2 which, together with vRNA and NP, forms a ribonucleoprotein (RNP) complex (Portela & Digard, 2002). PB1 is the polymerase, while PB2 binds the cap structures of cellular pre-mRNAs, which are then cleaved off through PA endonuclease function to prime viral mRNA synthesis (Boivin *et al.*, 2010). RNA synthesis takes place in the cell nucleus and involves multiple interactions with cellular molecules that act as both positive and negative factors for the overall process of virus replication (Josset *et al.*, 2008). It is plausible that some of these interactions contribute to host specificity and, consistent with this, much genetic evidence implicates the virus polymerase in defining host range (HR) (Naffakh *et al.*, 2008).

In particular, PB2 has long been known to play an important role in setting host specificity (Almond, 1977). Subsequent work showed that an avian virus could gain replication competence in mammalian cells through changing a single amino acid at position 627 of PB2 (Subbarao *et al.*, 1993). This has since been confirmed for many virus strains in cell culture and laboratory animals, as well as correlating with human disease (Hatta *et al.*, 2001; Le *et al.*, 2009; Munster *et al.*, 2007; Naffakh *et al.*, 2008). Until recently, most human viruses possessed a lysine (K) at position 627, while avian viruses have glutamic acid (E) (Chen *et al.*, 2006; Finkelstein *et al.*, 2007; Miotto *et al.*, 2008). However, PB2 627K is not obligatory for efficient infection or disease induction in mammals. Exceptions to the ‘627 rule’ include equine influenza viruses, some swine viruses, certain avian H5N1 isolates and, most notably, the 2009 pandemic virus, all of which have PB2 627E (Gao *et al.*, 1999; Schnitzler & Schnitzler, 2009; Shinya *et al.*, 2007). Indeed, engineering a 627K change into the 2009 pandemic virus did not result in increased virulence (Herfst *et al.*, 2010; Jagger *et al.*, 2010; Zhu *et al.*, 2010). In such cases, other residues within PB2 contribute to adaptation to replication in mammalian cells; such polymorphisms include T271A, A588I, GQ590/591SR, Q591K, D701N, S714R and probably others (Bussey *et al.*, 2010; Gabriel *et al.*, 2005; Li *et al.*, 2005, 2009; Mehle & Doudna, 2008, 2009; Naffakh *et al.*, 2000; Yamada *et al.*, 2010; Yao *et al.*, 2001).

The mechanism(s) by which alterations to the PB2 sequence extend HR is uncertain. Poor replication in mammalian cell culture is often correlated with low polymerase activity, especially in ‘minireplicon’ assays that measure viral transcription indirectly (Gabriel *et al.*, 2005; Labadie *et al.*, 2007; Massin *et al.*, 2001; Munster *et al.*, 2007; Naffakh *et al.*, 2000; Yao *et al.*, 2001). This plausibly reflects functionally important sequence-dependent interactions between PB2 and a cellular factor. Consistent with this, structural analysis of the C-terminal region of PB2 shows that residue 627 is surface exposed and that the E/K polymorphism makes a large difference to the charge of the domain face (Kuzuhara *et al.*, 2009; Tarendeau *et al.*, 2008). However, the identity of the putative HR-defining cellular protein(s) is unknown and, although many interaction partners of the viral polymerase have been identified, to date there are no reports of any that show 627E/K dependency. A nuclear localization signal (NLS) subdomain at the very C terminus of PB2 contains the HR-specific residues 701 and 714 (Mukaigawa & Nayak, 1991; Tarendeau *et al.*, 2007), and there is evidence of host-specific interactions with some importin α isoforms (Boivin & Hart, 2011; Gabriel *et al.*, 2008; Resa-Infante *et al.*, 2008). However, it is not clear that this mechanism explains the role of residue 627. Further confounding the issue, studies utilizing fusions of human and avian cells have come to conflicting conclusions about how the putative 627-dependent interaction affects polymerase function. One study favoured a human cell inhibitory factor that interacts with a 627E polymerase (Mehle & Doudna, 2008), while another proposed an activating factor that only acts on the 627K polymerase (Moncorgé *et al.*, 2010).

Here, we applied the live cell microscopy system recently established in our laboratory (Loucaides *et al.*, 2009) to investigate the effects of PB2 HR mutations on the nuclear dynamics of the viral polymerase. In support of the existence of a mammalian-specific interaction partner, we find that PB2 polymorphisms affect the dynamics of the viral polymerase in living cells, with low polymerase activity correlating with slow diffusional mobility. These data have implications for future strategies to identify the host factors involved.

## Results

cDNA clones from two virus strains were used to investigate the effects of PB2 HR mutations on the dynamics of the viral polymerase: A/PR/8/34 (PR8), a laboratory-adapted human isolate that retains mammalian signature residues at a majority of known loci (de Wit *et al.*, 2004; Fields & Winter, 1982), and A/turkey/England/50-92/91 (50-92), a highly pathogenic avian influenza virus that replicates poorly in mammalian cells and whose PB2 (hereafter, tPB2) has avian signature sequences (Howard *et al.*, 2007; Moncorgé *et al.*, 2010; Wood *et al.*, 1993). For both PB2 clones, ‘avianizing’ or ‘humanizing’ mutations were introduced into position 627, producing the set of PB2 variants PR8 wt, PR8 627E, tPB2 wt and tPB2 627K.

**Fig. 1.**
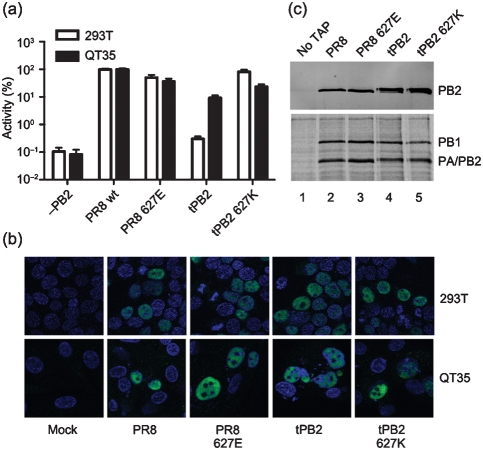
Functional characterization of PB2 627 variants. (a) Minireplicon activity assays were performed at 48 h in 293T (open bars) or QT35 (filled bars) cells. Readings were normalized (%) to PR8 wt PB2 levels. Values plotted are means±sem (*n* = 6–8). (b) 293T or QT35 cells were transfected with the indicated PB2 protein, fixed at 24 h and stained for PB2 in green; DAPI (blue) was used to stain nuclei. (c) SDS-PAGE and Western blot (top panel) and autoradiography (bottom panel) analysis of polymerase complexes purified over IgG-Sepharose. 293T cells were transfected as indicated with PB1–TAP (or untagged PB1; ‘no TAP’ lane), PA and PB2 and labelled with ^35^S-Met for 6 h at 24 h post-transfection before purification.

First, the influence of position 627 on viral polymerase activity was tested in minireplicon assays in human 293T and avian QT35 cells. PR8 PB1, PA, NP and the various PB2 plasmids were cotransfected with a plasmid expressing a synthetic vRNA encoding luciferase (under either a mammalian or avian RNA pol I promoter as appropriate) to reconstitute RNPs. Wild-type PR8 RNPs resulted in a high luciferase readout in both 293T and QT35 cells, while omitting PB2 reduced luciferase levels by 3 log_10_ to background values (Fig. 1a). Replacing PR8 PB2 with tPB2 decreased the activity of the viral polymerase several hundredfold in 293T cells while, in QT35 cells, the decrease was less than tenfold. Introducing the humanizing 627K mutation into tPB2 recovered polymerase activity to close to PR8 wt levels in both cell types. In contrast, introducing the avianizing 627E change into PR8 PB2 had little effect on polymerase activity in either cell type. Similar overall outcomes were obtained when the experiments were repeated in human HeLa and avian DF1 cells, as well as when RNPs were reconstituted using an authentic segment 8 vRNA or with GFP-tagged PB1 or PA subunits (data not shown). Therefore, while the single amino acid change in tPB2 had a major impact on viral transcription in human but not avian cells (as seen previously; Massin *et al.*, 2001; Moncorgé *et al.*, 2010), the introduction of the avian 627E residue in PR8 did not recapitulate this. The cell type- and position 627-dependency of tPB2 activity in the background of an otherwise PR8 RNP also indicates that there is no fundamental incompatibility between the avian PB2 and the mammalian-origin PB1, PA and NP proteins.

To test whether impaired transcription by tPB2 was a result of defective nuclear import in 293T cells, PB2 localization was examined by immunofluorescence. However, all PB2 variants localized efficiently to the nucleus of both 293T and QT35 cells, with no observable differences in staining pattern resulting from altering residue 627 (Fig. 1b). To test whether impaired polymerase function resulted from an inability to form 3P complexes in human cells, tandem affinity purification (TAP)-tagged purification assays were performed, where PB1–TAP (Engelhardt *et al.*, 2005), PA and the various PB2 constructs were transfected and cells were radiolabelled for 6 h at 24 h post-transfection. TAP-tagged complexes were purified on IgG Sepharose beads and cleaved off with TEV protease before analysis by SDS-PAGE. Similar amounts of PB1 and PA/PB2 were detected by autoradiography and, when Western blots for PB2 were performed, no obvious differences in 3P complex formation were observed (Fig. 1c).

**Fig. 2.**
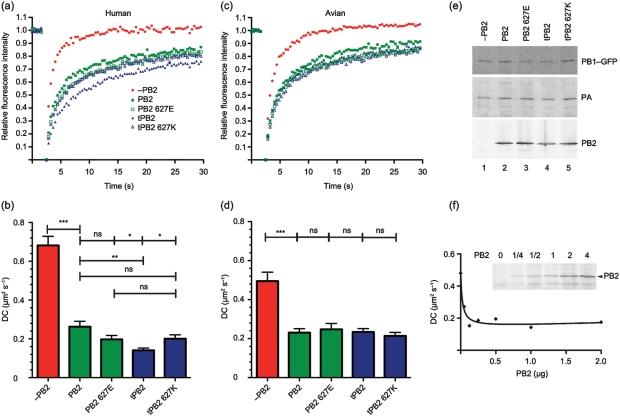
FRAP analysis of PB2 627 variants. (a, c) Average FRAP recovery curves of 3P complexes tagged with PB1–GFP in 293T cells (a) or QT35 cells (c) are plotted. (b, d) Mean±sem DC values measured in 293T (b) or QT35 (d) cells. Braces indicate significance tests: ns, non-significant; *, *P*<0.05; **, *P*<0.01; ***, *P*<0.001. (e) 293T samples were analysed by Western blotting for GFP, PA or PB2. (f) 293T cells were transfected with PB1–GFP, PA and the indicated amounts of PB2 plasmid (as fold difference from the other P proteins, with ‘1’ indicating 500 ng) and analysed by FRAP and (inset) Western blotting for PB2. The curve fit (*r*^2^ = 0.95) is for a one-site binding model.

Having validated the system, we applied the live cell microscopy technique of fluorescence recovery after photobleaching (FRAP) to examine the mobility of the viral polymerase complex in the nucleus of transfected cells. The hypothesis that restriction of polymerase complexes with an avian-like PB2 in 293T cells results from differential interactions with a mammalian host factor might be predicted to result in 627-dependent variations in polymerase dynamics. Accordingly, 3P complexes were reconstituted in cells by expressing PB1–GFP together with PA and the various PB2 proteins in either 293T or QT35 cells. When FRAP analyses were performed in 293T cells, as shown previously (Loucaides *et al.*, 2009), an incomplete polymerase complex consisting of only PB1 and PA showed rapid and almost complete recovery kinetics from the bleach phase, while the inclusion of PR8 wt PB2 resulted in a marked increase in the immobile fraction, as defined by the recovered fluorescence reaching a plateau at less than 100 % of the starting value (Fig. 2a; Table 1). The kinetics of this incomplete recovery also showed a marked decrease in the diffusion coefficient (DC) of the trimeric polymerase (Fig. 2b; Table 1). A similar pattern of significantly slower diffusion and increased immobile fraction of the complete 3P complex compared with the 2P dimer was obtained when the experiments were performed in avian QT35 cells (Fig. 2c, d), although the mobility of the PB1–GFP–PA dimer was slower in the avian cells than in the mammalian cells (Table 1). When PR8 PB2 was replaced by tPB2, the DC of the polymerase in 293T cells showed a significant decrease of nearly twofold compared with the mammalian-adapted polymerase, and the immobile fraction increased to over a quarter of the total population (Fig. 2a, b; Table 1). However, altering residue 627 in tPB2 to lysine significantly increased polymerase complex diffusion and reduced the immobile fraction to levels closer to the PR8 wt complex. ‘Avianizing’ PR8 PB2 with a 627E change had less effect on polymerase dynamics than complete replacement with tPB2, causing an increase in the immobile fraction and a decrease in the DC that was not statistically significant in comparison with the parental complex. When tested in avian QT35 cells, neither of the paired E627K changes altered mobility of the polymerase significantly (Fig. 2c, d; Table 1). Similar overall outcomes were obtained when the dynamics of PR8, tPB2 and tPB2 627K complexes were examined using PA–GFP in place of PB1–GFP (data not shown). Thus, the intranuclear dynamics of the viral polymerase complex correlate with transcriptional activity, with the identity of PB2 627 strongly affecting both measures when tPB2 was assessed in mammalian but not avian cells, and having less effect on the PR8 PB2 subunit. This is consistent with the hypothesis that the nature of residue 627 of an avian virus PB2 differentially affects protein–protein interactions in the nucleus of human cells.

**Table 1.** Effect of varying PB2 on the nuclear dynamics of the influenza virus polymerase The ‘Background’ column identifies the strain background and the identity of position 627, while the ‘Further mutations’ column indicates the presence or absence of further mutations. nd, Not done.

PB2 plasmid		293T			QT35		
Background	Further mutations	DC (μm^2^ s^−1^) (mean±sem)	Immobile fraction (mean±sem)	*n* (N)*	DC (μm^2^ s^−1^) (mean±sem)	Immobile fraction (mean±sem)	*n* (N)*
−PB2	–	0.68±0.05	0.01±0.01	31 (3)	0.49±0.05	0±0.03	46 (5)
PR8	wt	0.26±0.03	0.18±0.02	29 (3)	0.23±0.02	0.11±0.02	43 (5)
	588 636 701	0.20±0.03	0.20±0.02	29 (3)	nd	nd	nd
PR8 627E	wt	0.20±0.02	0.20±0.01	26 (3)	0.25±0.03	0.17±0.02	31 (3)
	588 636 701	0.14±0.01	0.25±0.02	31 (3)	nd	nd	nd
tPB2	wt	0.14±0.01	0.26±0.02	17 (2)	0.23±0.02	0.16±0.02	47 (5)
	588 636 701	0.16±0.01	0.26±0.01	31 (3)	nd	nd	nd
tPB2 627K	wt	0.20±0.02	0.21±0.01	25 (3)	0.21±0.02	0.19±0.02	46 (5)
	588 636 701	0.35±0.02	0.12±0.01	32 (3)	nd	nd	nd

* Total number of cells analysed from (in parentheses) the indicated number of independent transfections.

To confirm expression of the viral proteins, Western blots were performed on cells after FRAP analysis and levels were found to be similar between the samples (Fig. 2e). To test the potential significance of small fluctuations in PB2 levels on the mobility of the polymerase complex, we titrated PR8 wt PB2 in the presence of constant amounts of PB1–GFP and PA. Western blot analysis confirmed that transfection of increasing amounts of PB2 plasmid resulted in increasing accumulation of the polypeptide (Fig. 2f, inset). Parallel FRAP analysis showed that even the smallest amount of PB2 resulted in the characteristic drop in diffusional mobility of the PB1–GFP–PA dimer and that there was essentially no change in the DC over a close to tenfold range of plasmid dose (Fig. 2f). Thus, PB2 concentrations were not limiting and the FRAP therefore reflected intrinsic differences in the interactions of the PB2 variants, rather than small fluctuations in PB2 levels.

### Further HR-defining residues in PB2

**Fig. 3.**
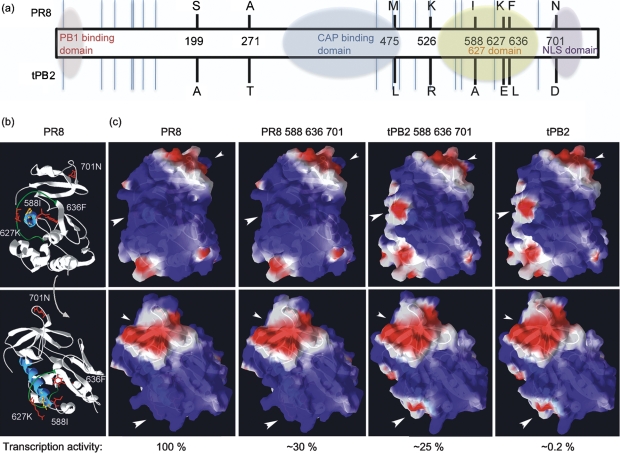
Location of PB2 HR-determining residues. (a) Linear representation of PB2 showing regions with structural information (shaded ovals: Guilligay *et al.*, 2008; Kuzuhara *et al.*, 2009; Sugiyama *et al.*, 2009; Tarendeau *et al.*, 2007) and the location of sequence polymorphisms between PR8 PB2 and tPB2 (black lines = tested, blue lines = non-tested). (b) Two views of a ribbon structure of the PR8 627 domain (3CW4 in PDB) with the side chains of mutated residues here shown in red. The 627 loop is green and the helix it encircles is blue. The side chains of residues G590 and Q591 are yellow. (c) Surface electrostatic potential modelling (using Swiss-Prot: red indicates negative charge, blue positive charge) on the 627 domain of PR8 or tPB2 (adapted from PDB 3L56; Yamada *et al.*, 2010). Large arrowheads indicate the 627 patch, small arrowheads the 701 patch. Values underneath are minireplicon activity in 293T cells.

**Fig. 4.**
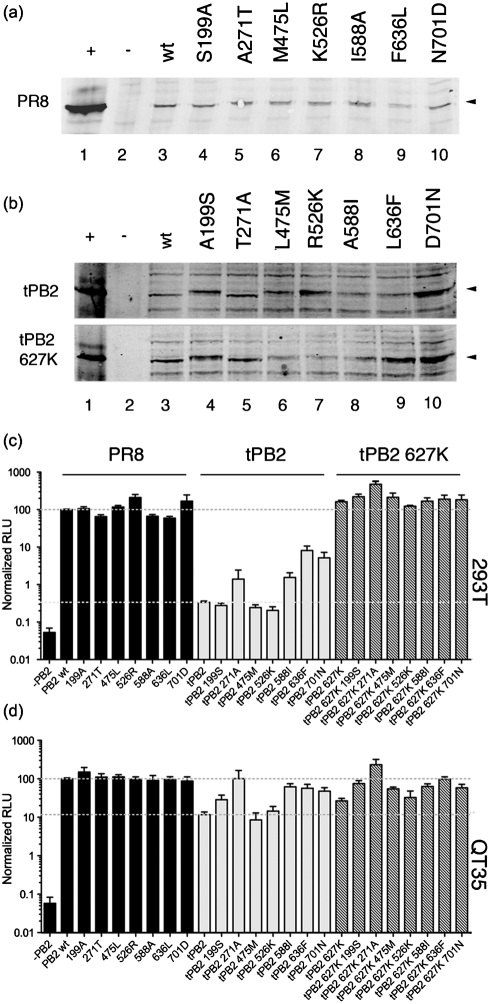
Expression and activity of PB2 HR mutants. (a, b) Western blot analysis of 293T cells transfected with the indicated PB2 polypeptides (−, no plasmid; +, infected cell lysate). (c, d) Minireplicon assay of HR mutants on PR8, tPB2 or tPB2 627K backgrounds in 293T (c) or QT35 (d) cells. Values are normalized to PR8 wt and dashed lines indicate this (upper) and tPB2 (lower) levels. Means±sem from at least three independent experiments are plotted. RLU, Relative luciferase units.

While the polymerase activity of the avian tPB2 in 293T cells exhibited a marked dependency on residue 627, introducing the 627E mutation into PR8 PB2 had only a minor effect (Fig. 1a). We therefore investigated whether other amino acid positions in the PB2 proteins affected their activity in a host-specific manner. Aligning tPB2 with PR8 PB2 revealed 24 differences (not shown) including, in addition to the E627K polymorphism, several other residues previously implicated by bioinformatics as avian/mammalian host-specific signatures (Chen *et al.*, 2006; Finkelstein *et al.*, 2007; Miotto *et al.*, 2008; Naffakh *et al.*, 2000). We chose a subset of these residues spread throughout the protein (summarized in Fig. 3a) for mutagenic analysis: A199S, T271A, L475M, R526K, A588I and D701N. We also investigated the L636F polymorphism, as it lies near the base of the loop structure that contains residue 627 and the helix where HR-related residues 588, 590 and 591 lie (Fig. 3b). Mutations were introduced into PR8 PB2, tPB2 and tPB2 627K backgrounds and the polypeptides were tested in minireplicon assays in 293T and QT35 cells. Western blot analysis confirmed expression of the proteins in 293T cells, although some of the point mutations caused slight mobility changes in the tPB2 polypeptides (Fig. 4a, b). Mutating PR8 PB2 had little effect on transcriptional activity in either mammalian or avian cells, making no more than a twofold difference (Fig. 4c, d). In contrast, humanizing tPB2 at positions 271, 588, 636 or 701 showed partial compensation for the lack of 627K in an otherwise fully avian PB2 in human cells, with changes at 636 and 701 increasing activity more than tenfold (Fig. 4c). However, these mutations also substantially increased the transcriptional activity of tPB2 in QT35 cells, although here, position 271 had the greatest effect (Fig. 4d). The 199, 271, 588, 636 and 701 changes also showed additive improvements in the transcriptional activity of tPB2 627K in QT35 cells, but only the 271 change had a substantial effect in mammalian cells. Thus, ‘humanizing’ changes to several of the potential HR-determining residues in tPB2 modulated its activity, albeit often (by smaller amounts) in avian as well as mammalian cells. However, ‘avianizing’ PR8 at these positions had little effect.

**Fig. 5.**
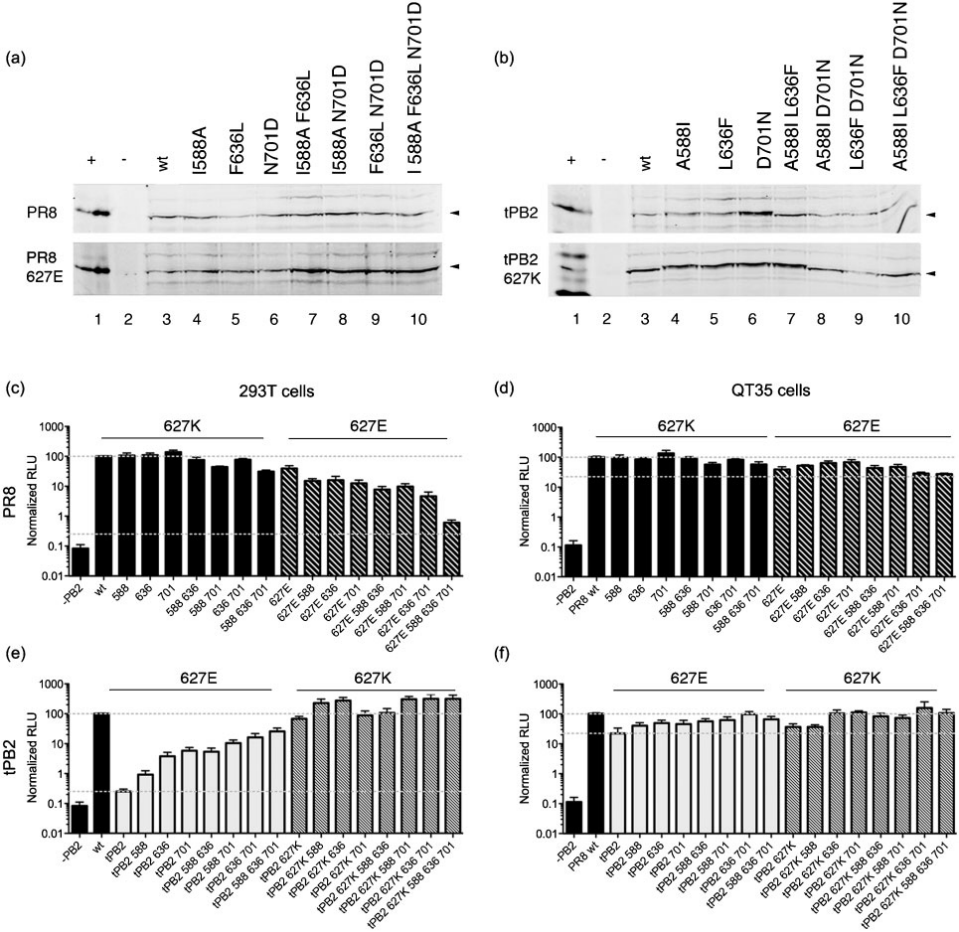
Synergistic effects of 627 domain HR mutations. (a, b) Western blot analysis of 293T cells transfected with the indicated PB2 polypeptides (−, no plasmid; +, infected cell lysate). (c–f) Minireplicon assay of HR mutants on PR8, PR8 627E, tPB2 or tPB2 627K backgrounds in 293T (c, e) or QT35 (d, f) cells. Values are normalized to PR8 wt and dashed lines indicate this (upper) and tPB2 (lower) levels. Means±sem from at least three independent experiments are plotted.

The 588, 636 and 701 polymorphisms are all in the 627/NLS domain of PB2 (Fig. 3), and we therefore investigated whether they exerted their effects synergistically, perhaps compensating for 627E in mammalian cells. Accordingly, the mutations were combined on the four backgrounds of PR8 wt, PR8 627E, tPB2 and tPB2 627K, before testing in minireplicon assays in human and avian cells. Parallel Western blot analysis confirmed that all polypeptides were expressed in 293T cells, with only minor variations in level (Fig. 5a, b). Even in combination, the additional mutations had relatively small effects (less than tenfold) in 293T cells when introduced onto a 627K background, either in PR8 PB2 or humanized tPB2 627K (Fig. 5c, e). They also had similarly small effects on the function of either PB2 protein in QT35 cells irrespective of the identity of position 627 (Fig. 5d, f). However, when tested in human cells on the background of 627E PB2, the additional mutations produced clear synergistic effects. Further avianizing PR8 627E with any of the mutations at positions 588, 636 and 701 reduced transcriptional activity nearly tenfold, while combining 636 with either of the 588 or 701 changes produced a greater than tenfold effect. Combining all four changes reduced PR8 PB2 activity in 293T cells almost to the level of wt tPB2. Conversely, adding additional humanizing mutations onto a tPB2 background incrementally increased polymerase activity in 293T cells even without the compensatory 627K residue. A triple-mutant tPB2 with 588I, 636F and 701N changes had activity more than a hundredfold greater than that of the parental tPB2, reaching levels only fourfold lower than PR8 wt PB2. Thus, PB2 amino acids 588, 636 and 701 also act as HR-defining residues that, together, compensate for glutamic acid at position 627.

**Fig. 6.**
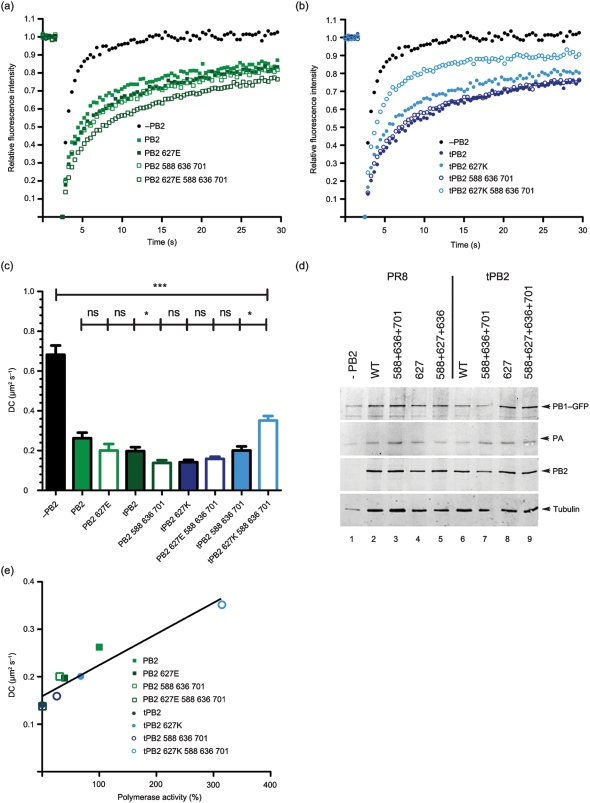
FRAP analysis of combined 627 domain HR mutations. (a, b) Average FRAP recovery curves of 3P complexes tagged with PB1–GFP in 293T cells for PR8 PB2 mutants (a) or tPB2 mutants (b). (c) Mean±sem DC values measured in 293T cells. Braces indicate significance tests: ns, non-significant; *, *P*<0.05; ***, *P*<0.001. (d) Cell lysates were analysed by Western blotting for PB1, PA and PB2. (e) Scatter plot of diffusion coefficient versus minireplicon activity in 293T cells. The line shows a linear curve fit.

To investigate whether, like residue 627, these additional PB2 HR mutations affected mobility of the polymerase complexes in human cells, we investigated their dynamics in 293T cells with FRAP. Adding the triple avianizing mutations onto either PR8 wt PB2 or PR8 627E caused slower recovery of the polymerase complexes and an increase in the size of the immobile fraction, to a point where the quadruple PR8 mutant behaved identically to tPB2 (Fig. 6a, c; Table 1). Adding the three humanizing mutations A588I, L636F and D701N onto tPB2 did not alter the immobile fraction of the polymerase complexes, but led to a slight increase in DC (Fig. 6b, c; Table 1). However, combining the humanizing mutations with the already humanized tPB2 627K resulted in a marked decrease of the immobile fraction and increase in DC even compared with PR8 wt PB2. Western blot analysis confirmed approximately equal levels of expression of the viral polypeptides (Fig. 6d). Therefore, other HR polymorphisms in the PB2 627 domain affect the dynamics of the polymerase complex in human cells, with mutations that reduce transcriptional activity in the context of an RNP also reducing diffusional mobility of the 3P complex. To explore the correlation between the two metrics, we plotted polymerase minireplicon activities against DC for the HR variants. Supporting a positive relationship between viral gene expression and polymerase mobility, a straight line fitted to the scatter plot showed a good correlation (*r*^2^ = 0.90; Fig. 6e).

## Discussion

Many studies have shown that PB2 residue 627 plays an important role in determining host restriction (Hatta *et al.*, 2001; Massin *et al.*, 2001, 2010; Steel *et al.*, 2009; Subbarao *et al.*, 1993), but the mechanism remains unclear. A plausible hypothesis invokes a mammalian-specific interacting protein, but it is controversial whether this is an inhibitory factor that prevents a 627E-containing polymerase from operating properly (Mehle & Doudna, 2008) or an important co-factor that only complements activity of a 627K polymerase (Moncorgé *et al.*, 2010). Furthermore, 627-dependent binding of any cellular component has yet to be demonstrated.

Here we show, using live cell microscopy, that the mobility of the viral polymerase complex varies in human cells according to the identity of PB2 627 and other HR-defining residues, in correlation with transcriptional activity. This correlation suggests that interactions of the viral polymerase with cellular factors influence its activity. However, it seems unlikely that slower polymerase mobility results directly from functional impairment, because we measured the diffusional dynamics under conditions where the enzyme is not active, lacking NP, and, furthermore, diffusion of the polymerase in our FRAP system is not affected by the presence of NP or RNP formation (Loucaides *et al.*, 2009). Also, the enzyme is active when immobilized on a solid support (Lee *et al.*, 2002, 2003). Instead, it seems more plausible that the twin phenomena of low activity and slow dynamics result from a common cause. Based on prior reports of 627-dependent defects in interactions between NP and the polymerase complex (Labadie *et al.*, 2007; Mehle & Doudna, 2008; Rameix-Welti *et al.*, 2009), we favour the hypothesis that low transcriptional activity results from a failure to form RNPs effectively, implying the involvement of host proteins in this assembly process. Our data are also consistent with suggestions that interactions between importin α and the NLS domain of PB2 affect the conformation and thus function of the polymerase in a host-specific fashion (Boivin & Hart, 2011; Resa-Infante *et al.*, 2008).

**Fig. 7.**
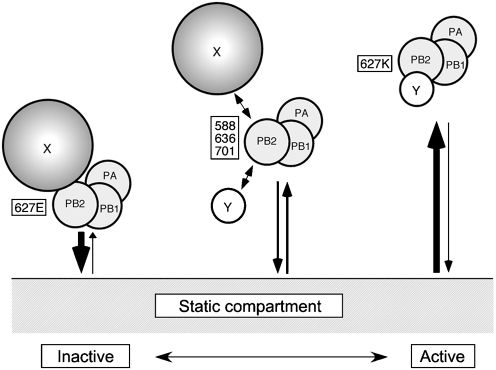
Models for HR-dependent changes in nuclear diffusion. Slow movement and low activity of 627E-containing polymerase could result from increased interactions with a relatively large soluble inhibitory cellular factor (‘X’) and/or with an insoluble ‘static compartment’. Higher mobility and activity of a 627K polymerase could result from decreased interactions with an inhibitor and/or increased affinity for a soluble activating factor (‘Y’). Intermediate-strength interactions occur with PB2 proteins containing compensatory mutations at other HR-affecting positions.

At the extreme, two alternative explanations for the slower diffusion of the less-active polymerase complexes can be envisaged. Either a 3P complex containing an inactive avian PB2 could be tightly bound to inhibitory cellular proteins that are sufficiently large to explain the reduction in mobility (Fig. 7; ‘X’), or the polymerase is tethered to an immobile nuclear component for longer periods than with a mammalian-adapted PB2 and therefore slower movement is observed (Fig. 7; ‘static compartment’). Within this framework, the hypothesis of an activating factor could envisage preferential binding of the mammalian 3P to a soluble cellular protein (Fig. 7; ‘Y’), which displaces another interaction to the static compartment and/or a larger inhibitory protein. In either case, the intermediate activities of PB2 proteins with changes to other HR-defining residues would reflect intermediate binding affinities. These hypotheses are not mutually exclusive, and it is possible that more than one activity-influencing host factor is involved. In agreement with previous work (Rameix-Welti *et al.*, 2009), however, we found no difference in the ability of our PB2/627 variants to bind cellular RNA polymerase II (data not shown).

While FRAP cannot distinguish between these hypotheses, the changing mobilities we observed for the 3P complex provide further experimental evidence for an interacting factor(s) that is (are) dependent on PB2 627. The interactions might be too transient to be detected readily in biochemical assays, but strong enough to influence the mobility and activity of the polymerase complexes. Our data and the models suggested in Fig. 7 have implications for strategies to identify the relevant interaction partners. The obvious route for identifying soluble activating or inhibitory factors is through examination of co-purifying proteins; here it may be useful to compare polymerase complexes at the extremes of activity and mobility, such as wt tPB2 and tPB2 mutated at positions 588, 627, 636 and 701. The finding that low activity correlates with slower mobility suggests that size-based fractionation techniques might be useful for focusing on an appropriate subpopulation of the viral polymerase. If the polymerase interacts with a static component of the nucleus, techniques adapted to deal with insoluble cellular components could be worthwhile.

While PB2 627 can act as a signature of avian versus mammalian viruses, some established mammalian viruses have a 627E residue (Gao *et al.*, 1999; Schnitzler & Schnitzler, 2009; Shinya *et al.*, 2007). Thus, while position 627 is often dominant, its importance is context dependent. Altering 627 in tPB2 made a more than 250-fold difference in 293T minireplicon activity, but had only a twofold effect on PR8 PB2 activity (Fig. 1a). Mutagenizing PR8 to resemble tPB2 and vice versa at positions 271, 588, 636 and 701 resulted in gradual convergence of the phenotypes (Figs 4 and 5). In agreement with Bussey *et al.* (2010), we found that altering positions 271 and 588 to the mammalian consensus improved polymerase activity. Residue 701 has been implicated previously in increased polymerase activity of a highly pathogenic avian influenza strain upon mammalian adaptation (Gabriel *et al.*, 2005). We also identified a novel PB2 residue important for HR activity, at position 636, where phenylalanine improved activity in mammalian cells in both virus backgrounds. This adds to a cluster of HR-defining residues formed by the N-terminal end of helix 5, the 627 loop and, now, β-sheet 1 (Fig. 3b; structural nomenclature as defined by Tarendeau and colleagues). F636 only occurs in a small minority of strains in the sequence database (as of January 2011, 13 of 8371 unique viruses) which, when PR8 vaccine derivatives and apparent instances of unnoticed PR8 contamination are discounted, leaves two other human isolates from the 1930s, A/Alaska/35 and A/Henry/36, and one swine isolate, A/swine/Tennessee/79/1977 (H1N1), all with a 627K residue. Therefore, either this polymorphism has not persisted (perhaps through lack of selective advantage in the presence of 627K) or it is a coincidental adaptation to laboratory passage. However, tPB2 polymerases containing it clearly displayed higher activity in human cells, so it will be interesting to see whether the sequence variation reoccurs in the future in 627E viruses.

In the swine-origin 2009 pandemic virus, residues 590/591 were identified as compensating positions for the lack of 627K (Mehle & Doudna, 2009; Yamada *et al.*, 2010); these residues lie close to position 588, which we found to be important in tPB2 and PR8 settings. However, while it has been proposed that the GQ590,591SR and Q591K polymorphisms function similarly to the E627K change in altering the electrostatic charge on one face of the 627 domain (Mehle & Doudna, 2009; Tarendeau *et al.*, 2008; Yamada *et al.*, 2010), our data argue against charge being the only determinant of function. A comparison of the predicted surface charge of the mammalian-active PR8 PB2 588, 636, 701 and tPB2 588, 636, 701 proteins with the inactive tPB2 protein strongly suggests that no obligatory correlation exists between electrostatic potential of the domain and transcriptional activity in mammalian cells (Fig. 3c). Thus, while we would not rule out an important charge-dependent interaction with a host protein, this seems unlikely to provide a full explanation of the role of the 627 and NLS domains in host restriction. While a single cellular protein whose binding is affected by the variety of HR mutations located in the 627 and NLS domains as well as elsewhere in PB2 is possible, multiple interactions (perhaps competitive) with cellular factors seem equally plausible. In favour of the former hypothesis, recent work has found evidence that binding of importin α to the PB2 NLS domain is subject to steric interference from the 627 domain, albeit not subject to the identity of residue 627 (Boivin & Hart, 2011). Consistent with the latter hypothesis, we have shown that a fragment of PB2 comprising the NLS domain and part of the 627 domain mediates competitive interactions with the viral NP and PB1 proteins (Poole *et al.*, 2004). Identification of the PB2-interacting cellular protein(s) responsible for setting HR will be key to resolving the mechanism of restriction.

## Methods

### 

#### Cell culture, plasmids and DNA transfection.

Human embryonic kidney 293T and quail QT35 cells were transfected using Lipofectamine 2000 according to the manufacturer’s instructions. Plasmids for PB1–GFP or PB1–TAP, pcDNA PB1, PA and NP as well as pPol-I-luc (with either a human or avian RNA pol I promoter) and pDUAL PR8 PB2 have been described elsewhere (Benfield *et al.*, 2008; de Wit *et al.*, 2004; Fodor & Smith, 2004; Mullin *et al.*, 2004). pDUAL plasmids for tPB2 and tPB2 627K were created by subcloning cDNA copies of the segments (Howard *et al.*, 2007) into the pDUAL vector (de Wit *et al.*, 2004). Site-directed mutagenesis was carried out using mismatched PCR primers and Phusion polymerase according to standard protocols. Primer sequences are available on request.

#### Protein and immunofluorescent analysis.

Antibodies used were mouse monoclonal anti-GFP JL-8 (Clontech), rat monoclonal γ-tubulin (YL1/2; AbD Serotec, MCA77G) and rabbit polyclonal anti-PB2 (2N580; Carrasco *et al.*, 2004). Western blots were developed using secondary antibodies conjugated to IR Dye 680 or 800 (LiCor Biosciences) and imaged on a LiCor Biosciences Odyssey instrument. Cells were stained for immunofluorescence and imaged with a Leica TCS SP confocal microscope as described previously (Wise *et al.*, 2009). Pull-down assays of TAP-tagged PB1 were performed as described by Fodor & Smith (2004). RNP reconstitution (‘minireplicon’) assays were carried out as described previously (Wise *et al.*, 2009). For FRAP analyses, 0.5 µg of each plasmid was transfected per dish.

#### FRAP microscopy.

Cells seeded on 42 mm coverslips were transferred to live cell chambers and maintained at 37 °C in Leibovitz L-15 medium (Invitrogen). Images were captured on a Zeiss LSM 510 confocal microscope using a ×63 NA 1.4 objective. Photobleaching was performed for 0.91 s using 100 % power from a 30 mW 488 nm laser line running at 6.1 mA, within a 1.94 µm^2^ square (ω^2^). Images were captured at 1 % laser output at 0.39 s intervals, obtaining 512×512 pixel images. Five pre-bleach and 70 post-bleach images were collected and the fluorescence intensities of regions of interest were obtained using the LSM510 software. Values were corrected by subtracting background fluorescence and then adjusted for whole nucleus intensity to account for fluorescence loss during the bleach period and acquisition photobleaching. Pre-bleach intensity was set to 1 and immediate post-bleach to 0 to allow comparison between samples. Data were fitted with a one-phase association model [*Y* = *Y*_0_+(*plateau*−*Y*_0_)·(1−*e*^−^*^K·x^*)] using Prism GraphPad [where *K* = ln(2)/*t*_1/2_ to yield *t*_1/2_ and plateau (*Y*_max_) values]. DCs were calculated from *t*_1/2_ values using the equation DC = 3.694(ω^2^/*t*_1/2_) according to the Axelrod model (Phair & Misteli, 2000). Student’s *t*-test (unpaired, two tailed; Prism GraphPad) was used to assess statistical significance.
